# P-996. Antibiotic Selection for Group B Streptococcus Prophylaxis in Penicillin Allergic Pregnant Patients

**DOI:** 10.1093/ofid/ofae631.1186

**Published:** 2025-01-29

**Authors:** Jenny Lam, YoungYoon Ham, Diana Yu

**Affiliations:** Oregon Health & Science University, Portland, Oregon; Oregon Health & Science University, Portland, Oregon; Doernbecher Children's Hospital/OHSU Healthcare, Portland, Oregon

## Abstract

**Background:**

Penicillin (PCN) is preferred by American College of Obstetricians and Gynecologists (ACOG) for the prevention of early-onset Group B Streptococcus (GBS infections in newborns, but 10% of patients report a PCN allergy. Clindamycin and vancomycin are second-line for GBS prophylaxis because they are less effective; thus ACOG recommends PCN testing in pregnant patients who report an allergy. Cefazolin has limited cross-reactivity in IgE-mediated PCN reactions and has excellent activity against GBS. This study aims to determine how PCN allergy testing is utilized in GBS positive, PCN allergic pregnant patients and its effect on antibiotic selection.

**Methods:**

This is a single-institution, IRB approved, retrospective cohort of patients that gave birth with an EHR documented PCN allergy from January 1, 2022-July 30, 2023. Pregnant patients over the age of 18 with a penicillin allergy and received antibiotics with an indication for GBS prophylaxis were included. Patients were excluded if they received antibiotics for indications other than GBS prophylaxis. The primary outcome was the rate of PCN used compared to cefazolin in skin-tested and non-skin-tested pregnant patients with indications for GBS prophylaxis. The secondary outcomes were rate of PCN allergy testing, adverse effects due to the antibiotic, the rate of neonatal early-onset sepsis, and frequency of PCN use compared to other antibiotics.

**Results:**

Of 4,780 births, 57 patients met inclusion criteria. Of those 57 patients, only 2 patients received PCN allergy testing referrals. Only 1 of the 2 PCN skin-tested patients had a negative result; however, both patients received cefazolin. The 55 patients without skin testing consults received penicillin (3.9%), cefazolin (51%), clindamycin (22%) and vancomycin (27.5%). Of the 25 patients who received clindamycin or vancomycin, all could have received cefazolin based on allergy cross reactivity data. There were no antibiotic-related adverse effects or neonatal early-onset sepsis.

**Conclusion:**

PCN allergy testing was found to be used very infrequently in this population; providers should be educated about the availability of this service. Additionally, providers should be educated regarding cross-sensitivity with PCN allergy and encouraged to use cefazolin for GBS prophylaxis.

**Disclosures:**

**YoungYoon Ham, PharmD**, Gilead: Honoraria

